# How Livelihood Change Affects Food Choice Behaviors in Low- and Middle-Income Countries: A Scoping Review

**DOI:** 10.1016/j.advnut.2024.100203

**Published:** 2024-03-09

**Authors:** Emma Kenney, Krystal K Rampalli, Sharraf Samin, Edward A Frongillo, Ligia I Reyes, Shiva Bhandari, Morgan Boncyk, Stella Nordhagen, Helen Walls, Sigrid Wertheim-Heck, Amy Ickowitz, Solveig A Cunningham, Ramya Ambikapathi, Beatrice Ekesa, Mirriam Matita, Christine E Blake

**Affiliations:** 1 Department of Health Promotion, Education, and Behavior, Arnold School of Public Health, University of South Carolina, Columbia, SC, United States; 2 Division of Nutritional Science, College of Human Ecology, Cornell University, Ithaca, NY, United States; 3 Department of Public Health, Purdue University, West Lafayette, IN, United States; 4 Global Alliance for Improved Nutrition (GAIN), Geneva, Switzerland; 5 Department of Global Health and Development, Faculty of Public Health and Policy, London School of Hygiene & Tropical Medicine, London, United Kingdom; 6 Environmental Policy Group, Department of Social Sciences, Wageningen University, Wageningen, The Netherlands; 7 Center for International Forestry Research-World Agroforestry Center, Beit Zayit, Israel; 8 Department of Global Health, Rollins School of Public Health, Emory University, Atlanta, GA, United States; 9 Department of Global Development, College of Agriculture and Life Sciences, Cornell University, Ithaca, NY, United States; 10 Alliance of Bioversity International and CIAT, Kampala, Uganda; 11 Extension Department, Lilongwe University of Agriculture and Natural Resources, Lilongwe, Malawi

**Keywords:** livelihood, food choice, scoping review, production, acquisition, preparation, distribution, consumption, occupation, locality, time, income, social relations

## Abstract

Livelihoods have changed dramatically over the past decade in low- and middle-income countries (LMIC). These shifts are happening in tandem with shifts in individual and household food choice behaviors. This scoping review aimed to identify and characterize mechanisms through which livelihood changes could affect food choice behaviors in LMIC, including behaviors relating to food production, acquisition, preparation, distribution, and consumption. A literature search was conducted using 4 databases: PubMed, PsycInfo, AGRICOLA, and Embase. The search was further enhanced by expert solicitations. Studies were included if they measured or focused on a livelihood change, described or assessed a change in ≥1 food choice behavior, and focused on LMIC. Studies were excluded if they focused on migration from LMIC to a high-income country. Of the 433 articles that were identified, 53 met the inclusion criteria. Five mechanisms of how livelihood change can affect food choice were identified: occupation, locality, time, income, and social relations. Changes in occupation altered the balance of the availability and affordability of foods in local food environments compared with individual food production. Changes in location, time use, and income influenced where food was purchased, what types of foods were acquired, and how or where foods were prepared. Additionally, changes in social relationships and norms led to expanded food preferences, particularly among urban populations. Time limitations and higher discretionary income were associated with consumption of ultraprocessed foods. Understanding the relationships between the changes in livelihood occuring in LMIC and food choices of households in these countries can inform the development of policies, programs, and other actions to promote sustainable healthy diets and planetary health.

**Statement of Significance**

This scoping review highlights 5 recurring mechanisms through which livelihood changes can impact food choice behaviors in low- and middle-income countries (LMIC), underscoring the intricate interplay between livelihood and diet. This insight can inform policies and programs to foster sustainable, healthy diets in LMIC, and promote planetary health.

## Introduction

What and how people eat is central to all cultures and intertwined with the livelihoods of the world’s populations. Over the past century, livelihoods globally have undergone transformations driven by technologic changes, social and economic factors, environmental influences, and individual choices, with rapid shifts over the last decade in Africa, Latin America, and Asia [1,2]. Although livelihoods can be defined broadly as a means of support or subsistence [3], for the scope of this article, livelihoods are defined as “activities that individuals participate in to acquire resources for securing necessities of life and to meet material and nonmaterial goals, including the maintenance or attainment of social status or membership in a particular group” [4].

People maintain their livelihoods through participation in formal and informal labor markets, as well as forms of passive resource-generating activities [5]. Labor markets consist of diverse employment arrangements characterized by particular schedules (e.g., shift work, full time, part time, and seasonal work), duration of work (e.g., fixed-term project or task-based occupations, casual or seasonal labor), geographic location (e.g., rural compared with urban, relocation patterns), compensation structure (e.g., hourly wages, daily wages, annual salaries, and piece work), employer–employee relations (e.g., supervisor–subordinate, self-employment, subcontracting, and temporary agency), and sector of the economy (formal or informal) [6]. In low- and middle-income countries (LMIC), over 75% of eligible adult workers are employed in the informal sector (e.g., occupations not subject to labor regulation, taxation, social protection, or benefits) [6]. Some livelihoods involve precarious conditions, including, intermittent work, more hours per week, lack of occupational safety and social protection, and limited free time [7]. Furthermore, work and income instability, particularly in the informal sector, disproportionally involve communities in poverty and add to the challenges faced in daily living.

Livelihoods change when individuals shift or adapt their means of supporting themselves and their households. This includes changes for people historically involved in food production. Globally, ∼1.23 billion people are employed in agrifood systems and 3.83 billion live in households linked to agrifood system-based livelihoods [8]. Employment in agrifood systems has declined in Africa from 60% in the early 2000s to 53% in 2019 and from 55% to 35% in Asia over the same period [8]. In many LMIC, recent large-scale livelihood changes have involved movement from on-farm to off-farm activities within food value chains, such as food processing, packaging, and distribution, or shifts out of food-related livelihoods entirely, or nonfarm activities such as mining or construction [9]. Livelihoods have also shifted from rural to urban areas, including the growth of secondary towns and cities where more employment opportunities are available [10]. Additionally, more females are seeking work opportunities outside the home, often alongside continued responsibility in domestic care and social reproduction [11].

Livelihood changes can dramatically influence the daily lives of individuals and households, impacting diets and human and planetary health through major alterations in individual and family food choice behaviors to changing food environment [[12], [13], [14], [15], [16], [17]]. Food choice is the decision-making process, both conscious and unconscious, through which individuals produce, acquire, prepare, distribute, and consume foods [18,19]. What, how, and why people eat the way they do must be understood within the biologic, economic, cultural, social, and environmental context in which the decision(s) are made [[18], [19], [20]]. Prior studies have demonstrated relationships between livelihood change and food choice behavior, but there is a lack of evidence on the mechanisms through which livelihood changes affect individual food choice behavior [21]. Livelihood changes may be forced or voluntary and are related to the power or agency of the people involved, resulting in both positive and negative outcomes. Forced changes are often to minimize risk from external factors (e.g., climate change, war, and disease), whereas voluntary changes are usually taken to invest in future opportunities [22,23]. These 2 reasons can have different implications for how households make food choice decisions.

This scoping review aimed to identify and characterize the mechanisms through which livelihood changes could affect food choice behaviors, including behaviors relating to food production, acquisition, preparation, distribution, and consumption. The review focuses on individual- and household-level changes rather than changes in food systems. Understanding the relationships between livelihood change and food choice can inform the development of policies, programs, and other actions to promote sustainable healthy diets and planetary health.

## Methods

The Drivers of Food Choice (DFC) Program was established to facilitate, synthesize, and disseminate research to provide a deeper understanding of what, how, and why people eat the way they do in Africa and Asia [24]. Over a 5-y period, the DFC program funded 15 projects in 10 countries, using a wide range of methods and led by diverse investigators from 38 collaborating institutions. Affiliates of the DFC program with expertise in food environments, food systems, and food choice research convened to identify priorities for evidence synthesis during the Agriculture, Nutrition, and Health Academy Week in Hyderabad, India, in June 2019. Livelihood characteristics were consistently identified as central to food choice. Findings from the DFC program portfolio guided initial conceptualization of the relationships between livelihoods and food consumption, and also informed the scope of this literature review [[25], [26], [27], [28], [29], [30]].

### Search strategy for the systematic scoping review

A systematic scoping review was performed using the PRISMA-ScR [31]. To identify potentially relevant articles, the following bibliographic databases were searched from November 2021 to July 2022: PubMed, PsycInfo, AGRICOLA, and Embase. Search strategies were drafted by coauthors (EK, KR, SS, CB) and refined through team discussion (Supplemental Table 1). The electronic database search was supplemented by expert solicitation (i.e., DFC technical advisory group, external DFC grantees, and well-known experts in the field) of articles from the DFC program and Google Scholar to expand the range of research articles and gather diverse perspectives.

### Inclusion and exclusion criteria

Articles were included if they met the following criteria: *1*) included ≥1 LMIC defined by the World Bank [32], *2*) measured or focused on a livelihood change, *3*) described or assessed a change in ≥1 food choice behavior (production, acquisition, preparation, distribution, and consumption), *4*) peer-reviewed, and *5*) published between 2015 and 2022. Quantitative, qualitative, and mixed-method studies using primary and secondary data were included. The year 2015 was used as a starting point because this is the year when the research community recognized the need to better understand food choice in LMIC, including through initiation of the DFC program [18,33,34]. Although the DFC program focused on Africa and Asia, articles from Latin-America were also included if they were set in countries defined as LMIC by the World Bank [32] between 2015 and 2022: Belize, Bolivia, El Salvador, Guatemala, Haiti, Honduras, and Nicaragua. Articles were excluded if they did not meet the criteria above or focused on migration from an LMIC to a high-income country.

### Data screening

**FIGURE 1:**
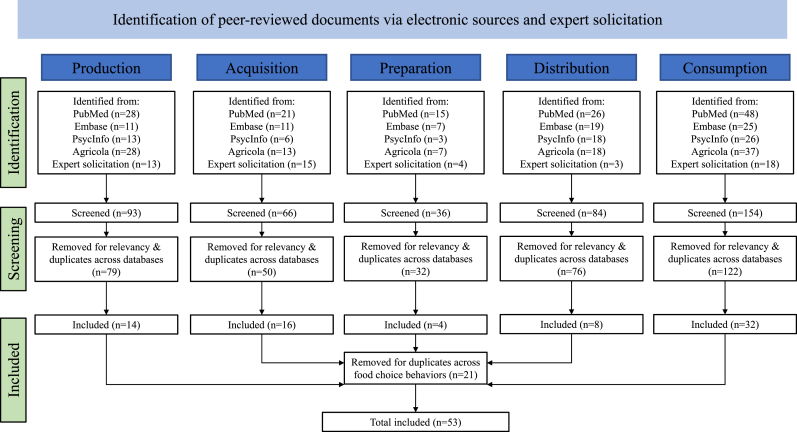
Flowchart of scoping review procedure for changes in food choice behaviors and livelihoods.

Articles were screened independently by 3 coauthors (EK, KR, and SS) for eligibility. Title and abstract screening were followed by full-text article screening. Duplicates were removed. After screening, the working group discussed the final articles for analysis (Figure 1).

### Data charting

A data-charting form was developed for data extraction. The following contents were extracted: title, first author, population, description of the livelihood change, mechanisms for how livelihood change affects food choice behaviors, description and dimension of the food choice behavior change (production, acquisition, preparation, distribution, and consumption). In this article, we define production as decisions about what and how food is grown or bred (for livestock, dairy, poultry, and fish) through agriculture, pastoralism, and/or aquaculture [35]. Acquisition is defined as food procurement through wild food gathering, production, market purchase, or through the social economy (e.g., bartering, gifting, direct monetary transactions) [36]. Preparation of food is the application of techniques (e.g., cleaning, chopping, and heating) to a combination of several foods or raw food materials to create a meal [37]. Distribution of food is how food is shared within a household among members [38]. Consumption is the process of ingesting food and involves eating episodes, including characteristics such as location and with whom [19].

The mechanisms of how livelihood changes could affect food choice behaviors were identified through qualitative thematic analysis of included studies [39]. Included articles were organized and independently charted by food choice behavior and reviewed to identify the mechanisms of livelihood change impacting each behavior. Coauthors (EK, KR, and SS) discussed results and iteratively updated the data-charting form. Additional coauthors were consulted when further clarification was needed. To ensure scientific rigor and reduce subjective bias, coauthors overlapped in the sections reviewed and reconciled interpretations [40]. When articles included >1 food choice behavior, each behavior was charted separately before combining all articles into 1 matrix. The final tabulation included each article once (*n* = 53), indicating which food choice behaviors were represented.

## Results

**FIGURE 2:**
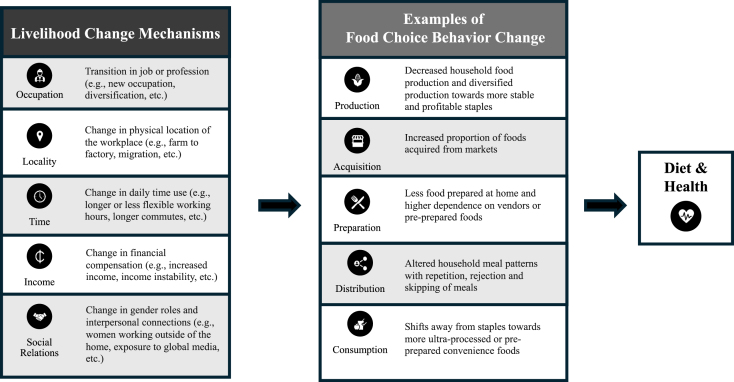
Indicates relationships between livelihood change mechanisms (occupation, locality, time, income, and social relations) and individual and household food choice behavior changes (production, acquisition, preparation, distribution, and consumption).

We identified 433 peer-reviewed articles through the database search (89%) and expert solicitation (11%). After reviewing the articles, 359 articles were excluded based on the review criteria (*n* = 295) and duplications (*n* = 64) across the 5 database searches. Articles were combined across food choice behaviors, excluding an additional 21 duplications. A total of 53 articles were included in the analysis (Table 1) [[25], [26], [27], [28], [29], [30],[41], [42], [43], [44], [45], [46], [47], [48], [49], [50], [51], [52], [53], [54], [55], [56], [57], [58], [59], [60], [61], [62], [63], [64], [65], [66], [67], [68]], from Africa (*n* = 25), Asia (*n* = 24), Latin America (*n* = 3), and mixed LMIC regions (*n* = 2). Sixteen were qualitative, 20 quantitative, and 17 were mixed methods. Of the studies with qualitative methods, most used in-depth interview and focus group discussion data collection approaches. Of those that used quantitative methods, 3 were longitudinal, 2 were randomized control trials, and all others were cross-sectional. Five recurring mechanisms of how livelihood changes affect food choice behaviors in LMIC were identified: occupation, locality, time, income, and social relations (Table 2 [[25], [26], [27], [28], [29], [30],[41], [42], [43], [44], [45], [46], [47], [48], [49], [50], [51], [52], [53],[55], [56], [57], [58], [59], [60], [61], [62],[64], [65], [66], [67], [68]] and Figure 2).

**TABLE 1:** Characteristics of included studies

Author (year of publication), and article title	Study design (e.g., cross-sectional, longitudinal)	Methodology (e.g., interviews, questionnaires, and focus group discussions)	Time period of data collection	Study area	Population
Sub-Saharan Africa (*n* = 25)					
Abera et al. [69] (2020). Social, economic and cultural influences on adolescent nutrition and physical activity in Jimma, Ethiopia: perspectives from adolescents and their caregivers	Cross-sectional	Qualitative: Focus group discussions	June–July 2018	Jimma, Ethiopia	Adolescents 10–12 y old and 15–17 y old (*n* = 41); parents (*n* = 22)
Ahishakiye et al. [41] (2021). Qualitative, longitudinal exploration of coping strategies and factors facilitating infant and young child feeding practices among mothers in rural Rwanda	Longitudinal	Qualitative: In-depth interviews	December 2016–April 2018	Rutobwe and Buramba health centers located in a rural part of Muhanga District, Rwanda	Mothers who followed recommended infant and young child feeding practices from birth to 1 y of child’s life (*n* = 17)
Amare and Shiferaw [42] (2017). Nonfarm employment, agricultural intensification, and productivity change: empirical findings from Uganda.	Longitudinal	Quantitative: Survey	2009–2012	Uganda	Households in national survey (*n* = 1846)
Arthur-Holmes and Busia [43] (2020). Household dynamics and the bargaining power of women in artisanal and small-scale mining in sub-Saharan Africa: a Ghanaian case study	Case study	Qualitative: In-depth interviews	July–September 2017	Prestea-Huni Valley Municipality of the Western Region, Ghana	Females involved in artisanal small-scale mining (*n* = 49)
Berhane et al. [44] (2018). Mixed blessings: a qualitative exploration of mothers’ experience of child care and feeding in the rapidly urbanizing city of Addis Ababa, Ethiopia	Cross-sectional	Qualitative: Semistructured interviews	March–November 2016	Urban Addis Ababa, Ethiopia (Lideta subcity)	Mothers with children under 5 y old (*n* = 36)
Bonye et al. [45] (2021). Urban expansion and agricultural land use change in Ghana: Implications for peri-urban farmer household food security in Wa Municipality	Retrospective	Mixed Methods: Questionnaires, interviews, historical GIS information	July 2019	Wa Municipality, Ghana	Smallholder farmers (*n* = 235)
Cockx et al. [46] (2018). From corn to popcorn? Urbanization and dietary change: evidence from rural-urban migrants in Tanzania	Longitudinal	Quantitative: Questionnaire	2008–2013	Tanzania	Rural–urban migrants (*n* = 238)
Ekesa et al. [25] (2020). Relationships between land tenure insecurity, agrobiodiversity, and dietary diversity of women of reproductive age: evidence from Acholi and Teso subregions of Uganda	Cross-sectional	Mixed Methods: Structured questionnaire	January–December 2017	Acholi and Teso subregions of Uganda	Females of reproductive age (*n* = 1227)
Hilson et al. [47] (2018). Female faces in informal ‘spaces’: women and artisanal and small-scale mining in sub-Saharan Africa	Case study	Qualitative: Interviews	Not specified	Sub-Saharan Africa with case studies in Sierra Leone and Zambia	Females involved in artisanal small-scale mining (*n* = not specified)
Irenso et al. [70] (2022). Maternal time use drives suboptimal complementary feeding practices in the El Niño-affected Eastern Ethiopia community	Cross-sectional	Qualitative: Focus group discussions	March–September 2016	Kersa district of eastern Ethiopia	Health Development Army leader, parent of child <2 y old, traditional birth attendant, or religious leader (*n* = 76)
Jodlowski et al. [48] (2016). Milk in the data: food security impacts from a livestock field experiment in Zambia	Cross-sectional	Quantitative: Survey	January 2012–August 2013	5 communities (Kamisenga, Kaunga, Kanyenda, Chembe, and Mwanaombe) from rural Zambia	Households (*n* = 324) in 5 communities (received livestock from Heifer International *n* = 3, did not *n* = 2)
Kibr et al. [71] (2020). Socio-economic variables associated with motivational barriers of food choice among lactating women from Central Ethiopia: a cross-sectional study	Cross-sectional	Quantitative: Face-to-face interviews using structured questionnaire	March–June 2016	Debrebirhan Town, Ethiopia	Lactating females aged 15–49 y(*n* = 423)
Manlosa et al. [72] (2019). Livelihood strategies, capital assets, and food security in rural Southwest Ethiopia	Cross-sectional	Mixed Methods: Survey questionnaire, field notes	November 2015–January 2016	6 kebeles (smallest administrative unit) in 3 districts, in Jimma Zone, Oromia Region, Ethiopia	Randomly selected households (*n* = 365) to capture variety of livelihood strategies
Matita et al. [26] (2021). Does household participation in food markets increase dietary diversity? Evidence from rural Malawi	Cross-sectional	Quantitative: Survey	May 2017–May 2018	Lilongwe and Phalombe districts of Malawi	Rural households (*n* = 400) interviewing “the person who makes the decisions about food preparation for the household”
Matitia et al. [73] (2022). Subsidizing improved legume seeds for increased household dietary diversity: evidence from Malawi’s Farm Input Subsidy Programme with implications for addressing malnutrition in all its forms	Longitudinal	Quantitative: Household survey	2013–2016	Malawi	Households in national survey (*n* = 2150)
Nikuze et al. [74] (2019). Livelihood impacts of displacement and resettlement on informal households—a case study from Kigali, Rwanda	Cross-sectional	Qualitative: Household interviews, focus group discussions, key informant interviews, field observations	May–September 2017	Kigali, Rwanda	Households (*n* = 185) in informal settlements to be displaced (*n* = 3) and resettlement sites (*n* = 3)
Nordhagen et al. [27] (2022). Between the city and the farm: food environments in artisanal mining communities in Upper Guinea	Cross-sectional	Mixed Methods: Market surveys, household surveys, observations, in-depth structured interviews	May 2018–December 2019	Artisanal gold mining communities in Kouroussa and Siguiri prefectures, Kankan region, Guinea	Mothers in mining households with children (survey *n* = 613; in-depth interview *n* = 45); food vendors (*n* = 40); single miners (*n* = 15)
Pace et al. [75] (2022). Cash transfers’ role in improving livelihood diversification strategies and well-being: short- and medium-term evidence from Zimbabwe	Randomized control trial	Quantitative: Survey	2013–2017	65 districts of Zimbabwe	Labor-constrained households living below the food poverty line (*n* = 3063)
Potts et al. [49] (2019). Animal source food consumption in young children from four regions of Ethiopia: association with religion, livelihood, and participation in the productive safety net program	Cross-sectional	Quantitative: Survey	October–December 2015	Afar, Amhara, Benishangul-Gumuz, and Tigray, Ethiopia	Parents/caretakers of children 0–36 mo old (*n* = 1009)
Ripkey et al. [28] (2021). Increased climate variability and sedentarization in Tanzania: health and nutrition implications on pastoral communities of Mvomero and Handeni districts, Tanzania	Longitudinal	Qualitative: Focus group discussions, in-depth interviews, key informant interviews, market surveys	February–October 2017	Mvomero and Handeni districts, Tanzania	Households (*n* = 60) in 6 villages (intensive sedentary *n* = 1, extensive sedentary *n* = 2, extensive pastoral *n* = 3); focus groups (*n* = 54); key informant interviews (*n* = 54)
Saronga et al. [50] (2016). “I eat two meals per day” impact of climate variability on eating habits among households in Rufiji district, Tanzania: a qualitative study	Cross-sectional	Qualitative: Focus group discussions	Not specified	Rural Rufiji district, Tanzania	Males and females aged 30–49 y who were residents of Rufiji district for >10 y (*n* = 90)
Sauer et al. [51] (2021). Consumption of processed food & food away from home in big cities, small towns, and rural areas of Tanzania	Cross-sectional	Quantitative: Survey	October 2011–October 2012	Tanzania	Households in national survey (*n* = 9788)
Stevano [52] (2017). The limits of instrumentalism: informal work and gendered cycles of food insecurity in Mozambique	Cross-sectional	Mixed Methods: Observation, structured and nonstructured interviews and life histories, household surveys	2011–2012	Pemba (urban), Metuge (periurban) and Mueda (rural) districts of Cabo Delgado province, Mozambique	Focus groups with females in agricultural associations, farmers, or traders (*n* = 31); survey of females in randomly selected households (*n* = 120); interviews with females doing paid work/cash earning activities (*n* = 60)
Stokes-Walters et al. [30] (2021). “If you don't find anything, you can't eat” – mining livelihoods and income, gender roles, and food choices in northern Guinea	Cross-sectional	Mixed Methods: Survey, in-depth interviews	May 2018*–*December 2019	Artisanal gold mining communities in Kouroussa and Siguiri prefectures, Kankan region, Guinea	Mothers in mining households with children (survey *n* = 613; in-depth interview *n* = 45)
Zolnikov [76] (2020). Effects of the government's ban in Ghana on women in artisanal and small-scale gold mining	Cross-sectional	Qualitative: Semistructured interviews	Not specified	Rural Akwatia, Ghana	Female miners affected by government ban of females in mining (*n* = 21)
Asia (*n* = 24)					
Alston and Akter [53] (2016). Gender and food security in Bangladesh: the impact of climate change	Cross-sectional	Mixed Methods: Interviews, focus group discussions, large quantitative survey	2011–2013	Ghaibanda, Satkhira, Barguna regions, Bangladesh	Males and females (surveys *n* = 617; interviews *n* = 33; focus groups *n* = 29)
Ashley et al. [54] (2018). Livestock and livelihoods of smallholder cattle-owning households in Cambodia: the contribution of on-farm and off-farm activities to income and food security	Cross-sectional	Quantitative: Survey	January–December 2016	Tramkok, Tbong Khmum, Banan, and Bantheay Srey, Cambodia	Cattle-owning households (*n* = 20)
Choithani [77] (2019). Gendered livelihoods: migrating men, left-behind women and household food security in India	Cross-sectional	Mixed Methods: Surveys, observations, interviews	January 2012–October 2013	Rural Siwan district, Bihar, India	Rural household surveys (migrants *n* = 197, nonmigrant *n* =195, left-behind wives of migrants *n* = 144)
Euler et al. [78] (2017). Oil palm adoption, household welfare, and nutrition among smallholder farmers in Indonesia	Cross-sectional	Quantitative: Face-to-face interviews using structured questionnaire	September–December 2012	Jambi province, Sumatra, Indonesia	Farm households (*n* = 664: oil palm adopters *n* = 199, nonadopters *n* = 465)
Gibson et al. [79] (2021). Coping or adapting? Experiences of food and nutrition insecurity in specialised fishing households in Komodo District, eastern Indonesia	Cross-sectional	Mixed Methods: Household survey, semistructured interviews, focus group discussions, key informant interviews	September 2017–May 2018	Komodo District, Indonesia	Household surveys with ≥1 female (18–49 y old) and a child (6 mo to 5 y old) (*n* = 125); Key informant interviews (*n* = 16); semistructured interviews (*n* = 24); focus group discussions (*n* = 9)
Gironde et al. [55] (2021). No cash, no food. Gendered reorganization of livelihoods and food security in Cambodia	Cross-sectional	Mixed Methods: Questionnaire-based surveys, semistructured interviews	January–May 2016	Ratanakiri, Kratie, and Kampong Thom provinces, Cambodia	Households (*n* = 600); farmers (*n* = 211); local authorities (*n* = 45)
Gurung et al. [56] (2016). Transformation from rice farming to commercial aquaculture in Bangladesh: implications for gender, food security, and livelihood	Cross-sectional	Mixed Methods: Focus group discussions, semistructured questionnaire	December 2011–April 2012	Khulna, Satkhira, and Mymensingh districts, Bangladesh	Farm households (*n* = 400: rice cultivation households *n* = 120, commercial aquaculture *n* = 280); focus groups (*n* = 10)
McCarthy [57] (2019). The paradox of progressing sideways: food poverty and livelihood change in the rice lands of outer island Indonesia	Cross-sectional	Mixed Methods: Qualitative interviews, household livelihood surveys	Not specified	Rice producing lowlands of Aceh in outer island Indonesia	Randomly selected households (*n* = 80)
Morrison et al. [80] (2018). Formative qualitative research to develop community-based interventions addressing low birth weight in the plains of Nepal	Cross-sectional	Qualitative: Semistructured interviews, focus group discussions	Not specified	Rural Dhanusha district, Nepal	Daughter-in-laws from marginalized groups living in extended families (*n* = 25)
Mottaleb et al. [81] (2017). Consumption of food away from home in Bangladesh: do rich households spend more?	Cross-sectional	Quantitative: Survey	2000–2010	Seven divisions and 64 districts of Bangladesh	Households in national survey (*n* = 29,648)
Naz et al. [58] (2020). Women's contribution in provision of household food security: a study from rural areas of Punjab, Pakistan	Cross-sectional	Quantitative: Survey	2018	Faisalabad, Vehari, and Chakwal districts of Punjab, Pakistan	Primary female (18–60 y old) in rural households (*n* = 420)
Piple et al. [82] (2015). Food choices and consequences for the nutritional status: insights into nutrition transition in an hospital community	Cross-sectional	Quantitative: Questionnaire, anthropometry	Not specified	India	Community hospital workers (*n* = 128)
Rowland et al. [29] (2022). Oil palm and gendered time use: a mixed-methods case study from West Kalimantan, Indonesia	Cross-sectional	Mixed Methods: Questionnaires, Focus group discussions, key informant interviews	January–August 2018	West Kalimantan, Indonesia	Households with children aged 12 mo to 5 y (*n* = 603 individuals)
Sahu [83] (2018). Household drought coping, food insecurity and women in Odisha	Cross-sectional	Quantitative: Field surveys	Not specified	Bolangir and Kendrapada districts, Odisha, India	Rural households in remote, drought prone areas (*n* = 76)
Seng [59] (2015). The effects of nonfarm activities on farm households’ food consumption in rural Cambodia.	Cross-sectional	Quantitative: Survey	2009	Cambodia	Rural farmers in national survey (*n* = 5762)
Shukri et al. [84] (2018). Relationship between work-family conflict and unhealthy eating: Does eating style matter?	Cross-sectional	Quantitative: Questionnaire	Not specified	Kuala Terengganu, Malaysia	Malaysian adults (*n* = 586: normal weight *n* = 437, overweight *n* = 149)
Sousa et al. [60] (2022). Patterns of street food purchase in cities from Central Asia	Cross-sectional	Quantitative: Direct observation	April–May 2016	Urban areas of Dushanbe (Tajikistan), Bishkek (Kyrgyzstan), Ashgabat (Turkmenistan) and Almaty (Kazakhstan)	Street food customers (*n* = 714)
Steenbergen et al. [61] (2017). Effects of rapid livelihood transitions: examining local co-developed change following a seaweed farming boom	Case study	Mixed Methods: In-depth interviews, focus group discussions, seaweed monitoring	2012–2016	Tanimbar Kei Island, Southeast Maluku, eastern Indonesia	Seaweed farming households, traders, and community leaders (*n* = 26); focus groups (*n* = 6)
Surendan et al. [85] (2020). Characterising the fruit and vegetable environment of peri-urban Hyderabad, India	Cross-sectional	Qualitative: In-depth interviews, focus group discussions	June–August 2017	9 periurban villages of Ranga Reddy district, Telangana	Households with ≥1 adult male and female (18–65 y old) (*n* = 18); focus groups (*n* = 9)
Tamrakar et al. [86] (2020). Drivers of healthy eating in a workplace in Nepal: a qualitative study	Cross-sectional	Qualitative: Focus group discussions, in-depth interviews	Not specified	Dhulikhel Hospital Kathmandu University Hospital (DH-KUH), Nepal	Hospital employees (*n* = 42)
Tran and Vu [62] (2020). The pro-poor impact of non-crop livelihood activities in rural Vietnam: a panel data quantile regression analysis	Cross-sectional	Quantitative: Survey	2008–2016	12 Provinces of Vietnam	Households in national survey (*n* = 2131)
Wertheim-Heck and Raneri [63] (2020). Food policy and the unruliness of consumption: an intergenerational social practice approach to uncover transforming food consumption in modernizing Hanoi, Vietnam	Cross-sectional	Qualitative: Interviews, direct observation	April–May 2018	Hanoi, Vietnam	Multigenerational households with 2 generations of females above 20 y old living together [daughters (the main respondent) and their mothers (in-law)] (*n* = 14)
Wertheim-Heck and Spaargaren [87] (2016). Shifting configurations of shopping practices and food safety dynamics in Hanoi, Vietnam: a historical analysis	Retrospective	Mixed Methods: Qualitative ethnography, quantitative census, desk research	1975–2015	Urban Hanoi, Vietnam	Vegetable shoppers (*n* = 50); households (*n* = 11)
Central and South America (*n* = 3)					
Cleary et al. [64] (2022). Changes in food consumption in an indigenous community in southern Belize, 1979–2019	Longitudinal	Mixed Methods: Surveys, semistructured interviews	1979–2019	Rural Aguacate district, southern Belize	Households recruited by the village schoolteacher (*n* = 17)
Kurschner et al. [65] (2020). Impact of school and work status on diet and physical activity in rural Guatemalan adolescent girls: a qualitative study	Cross-sectional	Qualitative: In-depth interviews	March–July 2018	Tecpán, Guatemala	Adolescent girls aged 15–19 y (*n* = 20) (in school and unemployed *n* = 8, in school and employed *n* = 4, not in school and employed *n* = 8)
Oddo et al. [66] (2018). Pathways of the association between maternal employment and weight status among women and children: qualitative findings from Guatemala	Cross-sectional	Qualitative: Semistructured interviews	February–May 2015	Nuevo San Carlos, Guatemala	Mothers enrolled in Project MIEL (*n* = 20)
Mixed region (*n* = 2)					
Banerjee et al. [67] (2015). A multifaceted program causes lasting progress for the very poor: evidence from six countries	Randomized control trial	Quantitative: Survey	2017–2014	Ethiopia, Ghana, Honduras, India, Pakistan, Peru	Households with extreme poverty based on a participatory wealth-ranking process(*n* = 10,495)
Diehl et al. [68] (2019). Household food consumption patterns and food security among low-income migrant urban farmers in Delhi, Jakarta, and Quito	Cross-sectional	Mixed Methods: Surveys, semistructured interviews	December 2017–June 2018	Urban areas of: New Delhi, India; Jakarta, Indonesia; Quito, Ecuador	Market-oriented small-to-medium scale farmers (*n* = 333)

**TABLE 2:** Summary of relationship between livelihood change and food choice behavior changes for included studies

Author(s) (year of publication), and article title	Livelihood change	Mechanisms of livelihood change					Examples of livelihood changes impacting food choice behaviors	Dimensions of food choice behavior change				
		O	L	T	I	SR		P	A	Pr	D	C
Africa (*n* = 25)												
Arthur-Holmes and Busia [43] (2020). Household dynamics and the bargaining power of women in artisanal and small-scale mining in sub-Saharan Africa: a Ghanaian case study.	• Agriculture/livestock rearing → informal artisanal mining (ASM) • Husbands/partners were taking care of children while females were at workplaces	X	X	X	X	X	• Female miners could bargain with their partners on dietary arrangements because they were contributing to the family food provision, mainly when their husbands could not afford the total expenses on food • Females’ relative economic resources increased their individual preferences toward certain food choices → able to afford better food, not just for themselves but also for their children, thus improving their nutritional well-being		X	X	X	X
Cockx et al. [46](2018). From corn to popcorn? Urbanization and dietary change: Evidence from rural-urban migrants in Tanzania.	• Rural–urban migration • Agriculture → nonfarm/informal/wage work	X	X	X	X	X	• Separation of living and work location and longer commuting distances in urban areas are assumed to raise the opportunity costs of time spent on acquiring and preparing food and induce greater preferences for more conveniently consumed and preprepared foods • Changes in preferences and habits arise in urban areas as a consequence of greater exposure to more global eating patterns, modern mass media, and improved access to formal or informal nutrition knowledge • Changes in income → increase in consumption of rice, high-sugar products, and meals and snacks consumed away from home		X	X		X
Nordhagen et al. [27] (2022). Between the city and the farm: food environments in artisanal mining communities in Upper Guinea.	• Females shift to artisanal and small-scale mining	X	X	X	X	X	• Households obtain limited food from own production and are largely dependent on markets • Mining directly influences preferences for foods and drinks seen to give energy—such as instant coffee or canned energy drinks • Time places an additional constraint on food access and further emphasizes convenience, as miners are working at the site from early morning until late afternoon—limited time for food preparation • Changes in mealtimes or meal skipping • Miners chose vendors based on ability to buy on credit because of income instability • Increased income → consuming (or increasing) fish, meat, or chicken; shifting from fish to meat; adding vegetables; increasing the amount of oil and having more sauce for a given amount of rice • Decrease income → no fish (or meat), less rice, no or different vegetables and using red palm oil instead of commercially processed vegetable oil	X	X	X		X
Sauer et al. [51] (2021). Consumption of processed food & food away from home in big cities, small towns, and rural areas of Tanzania.	• Urban females: no employment/homemaker → work outside the home (nonfarm wage labor or self-employment) • Rural females: on-farm → rural nonfarm employment (self-employment and/or wage employment)	X	X	X	X		• In rural areas 60% of food consumption comes from purchases in value terms, and processed food accounts for 76% of purchases and 47% of all food consumed. For the rural poor, purchased processed food is 38% of food consumption • In urban areas processed food’s share of purchases is 78%, similar for the rich and poor • Ultraprocessed food and meals away from home have emerged as important in urban and rural areas • The spread of processed food consumption in rural and urban areas, among the rich and poor, is driven mainly by opportunity costs of the time of females and males, and thus the pursuit of saving home-processing and cooking time, as well as food environment factors		X	X		X
Stevano [52] (2017). The limits of instrumentalism: informal work and gendered cycles of food insecurity in Mozambique.	• Females diversifying livelihoods through wage work and cash-generating activities, in addition to farming • Lack of regular employment resulting in unstable work and irregular income	X		X	X	X	• Poorly diversified diets overly dependent on cereals, vegetables, and legumes because of low and fluctuating incomes • Trade-off between working and preparing food • Reducing number of meals per day in response to time constraints • Repetition of meals may lead to refused foods because of being tired of having the same meal more than once in a day			X	X	X
Stokes-Walters et al. [30] (2021). “If you don't find anything, you can't eat” – mining livelihoods and income, gender roles, and food choices in northern Guinea.	• Females in agricultural → mining livelihood	X		X	X	X	• Food is purchased upon returning from the mining site → what is prepared for the evening meal is a reflection of a miner's daily success • If income is lacking, females usually skip meals themselves to ensure enough food for their families • Preparing and cooking meals remains a female responsibility, even when both males and females are engaged full time in work • Food vendors at the mining sites typically sold preprepared meals or “quick food items” • Typically eat later in the evening • Some females brought their children to the mining sites, whereas some left their children with older siblings → during their time at the mining site, more than half of the children were not fed. Other children of miners were fed snacks that could be purchased from nearby vendors	X	X	X	X	X
Zolnikov [76] (2020). Effects of the government's ban in Ghana on women in artisanal and small-scale gold mining.	• Nonfarm self-employment and informal employment (e.g., selling produce or cooked food) → small-scale artisanal mining • Transitions back to self-employment after government ban led to forced removal/displacement	X	X		X	X	• Many females were unable to pay for their children’s school fees, as well as other necessities of life, such as electricity, water, and food • Increases in food insecurity → eating less food per day		X			X
Berhane et al. [44] (2018). Mixed blessings: a qualitative exploration of mothers’ experience of child care and feeding in the rapidly urbanizing city of Addis Ababa, Ethiopia.	• Rural–urban migration for work • Construction/building sector provides one of the largest employment opportunities for unskilled labor • Forced displacement/relocation from familiar neighborhoods - resettlement dismantled social networks and affected their livelihoods • Mothers with family around to help them with childcare and those with the ability to get flexible working hours were said to have better capacity to engage in paid work		X	X	X	X	• Increased acquisition of unhealthy, processed foods because of time constraints • Mothers dependent on remittances sent to them by migrant husbands working abroad or elsewhere. The income was to enhance food purchasing ability but absence of the father forced the mothers be responsible for “everything else,” restricting her mobility as well as her engagement in social activities • Females take income earning jobs to support their families → deteriorated quality of care children receive in their absence (i.e., not being fed enough food, being fed unhygienic foods, etc.)		X		X	
Hilson et al. [47] (2018). Female faces in informal ‘spaces’: women and artisanal and small-scale mining in sub-Saharan Africa.	• Agriculture/livestock rearing → informal artisanal mining, nonfarm self-employment, or other informal sector jobs (domestic workers, industrial outworkers, etc.)	X	X		X		• Many females miners income from gold mining is used to purchase a wide range of fresh foodstuffs from nearby villages, including cassava, aubergines, oranges, limes and mangoes, which they would then transport to sell at much higher prices		X			
Ahishakiye et al. [41] (2021). Qualitative, longitudinal exploration of coping strategies and factors facilitating infant and young child feeding practices among mothers in rural Rwanda.	• Females working • Agriculture → income-generating activities such as selling handcrafts, raising animals, etc.	X		X		X	• Prioritized child feeding over livelihood chores • Livelihood diversification of mothers → prepared child’s food in advance			X	X	
Amare and Shiferaw [42] (2017). Nonfarm employment, agricultural intensification, and productivity change: empirical findings from Uganda.	• Shift to nonfarm employment	X		X	X		• Agricultural productivity declines as nonfarm income increases • Nonfarm activities divert time and effort away from agricultural activities and did not increase investments in productivity-enhancing agricultural technologies (e.g., fertilizer), which could have subsequent implications on agricultural productivity • Households that engage in nonfarm employment hire labor to undertake timely farm practices and some of the nonfarm income is used to finance the purchase of improved seeds	X				
Ripkey et al. [28] (2021). Increased climate variability and sedentarization in Tanzania: health and nutrition implications on pastoral communities of Mvomero and Handeni districts, Tanzania.	• Pastoralists → agro-pastoralism and wage labor/petty trade • Sedentarization • Increased number of large-scale farms • Females increasingly engaged in small business	X			X	X	• Increased drought → reduced availability of grazing land for cattle → decreased milk production and increases in cattle disease → decreases in highly liquid financial capital • Reduced number of meals per day and consumption of milk, meat, and wild produce • Increased consumption of novel foods including more processed foods such as refined maize meal and chips • Customary food sourcing, heavily based in livestock products, gathering wild foods, and some homestead crop cultivation → to greater dependence on staples and what is available and affordable in markets • Boiling meats and vegetables → frying with refined cooking oils	X	X	X		X
Abera et al. [69] (2020). Social, economic and cultural influences on adolescent nutrition and physical activity in Jimma, Ethiopia: perspectives from adolescents and their caregivers.	• Inward migration from rural areas for better paying jobs		X	X	X		• Shared meals to save time, labor, cost and resources • Family economic status as a strong driving force in determining adolescent diet				X	X
Saronga et al. [50] (2016). “I eat two meals per day” impact of climate variability on eating habits among households in Rufiji district, Tanzania: a qualitative study.	• Farming → wage work or other nonfarm income-generation activities (small businesses and cultivating on the valleys)	X			X		• Not enough food from their farms because of floods and droughts → shopped for food at retail outlets instead • Developed adaptation mechanisms that included reducing food quantity or eating of new meals which were not eaten before as a main meal such as cooked unripe mangoes and stiff porridge • Reduced number of meals, eating 2 meals a day instead of 3 or 4	X	X			X
Jodlowski et al. [48] (2016). Milk in the data: food security impacts from a livestock field experiment in Zambia.	• Shift to livestock production	X			X		• Increased consumption of animal products produced on farm and increased consumption expenditures	X				X
Pace et al. [75] (2022). Cash transfers’ role in improving livelihood diversification strategies and well-being: short- and medium-term evidence from Zimbabwe.	• Rural livelihood diversification through cash transfer • On-farm specialization vs. survival-led diversification vs. opportunity-led diversification vs. full diversification	X			X		• Increased food consumption for those that shift to opportunity-led and full diversification					X
Matita et al. [26] (2021). Does household participation in food markets increase dietary diversity? Evidence from rural Malawi.	• Engagement in nonagricultural income-generating activities • Engagement in agricultural intervention (FISP)	X			X		• Although the FISP has been implemented to promote nutrition-sensitive agriculture by providing legume seeds in addition to improved maize seed and fertilizer, this does not appear to have particularly affected food choices and dietary diversity in any significant way • Access to food markets was more important for dietary diversity than diverse farm production	X	X			X
Irenso et al. [70] (2022). Maternal time use drives suboptimal complementary feeding practices in the El Niño-affected Eastern Ethiopia community.	• Agricultural production → income-generating activities • Females frequently went to the nearest town/cities (Dire Dawa city and Kersatown) for petty trading, including selling firewood and charcoal produced locally • Males (older boys and fathers) worked as daily laborers, stayed and worked in urban areas, and only visit the family periodically	X		X			• Maternal time allocation for child feeding negatively influenced by staying away from home for work → low participation in community-based complementary feeding activities • Low level of direct male involvement influences the initiation of complementary foods, including the early provision of butter and sweets such as biscuits and the over-reliance on unmodified family foods at an early age • Effects on intrahousehold food distribution - Older children are eating more of the food meant for younger children				X	X
Kibr et al. [71] (2020). Socio-economic variables associated with motivational barriers of food choice among lactating women from Central Ethiopia: a cross-sectional study.	• More females in the workforce (i.e., government employee, day laborer, self-employee, merchant)	X				X	• Those that were self-employed reported greater religious influence for their food choice • Housewives reported greater influence of food ingredients on food choice					X
Ekesa et al. [25] (2020). Relationships between land tenure insecurity, agrobiodiversity, and dietary diversity of women of reproductive age: evidence from Acholi and Teso subregions of Uganda.	• Lord's Resistance Army civil war → displacement from land → conflicts over land and land boundaries • Land tenure secure vs. land tenure insecure		X		X		• Area of land farmed dropped 50% from an average of ∼8 acres to an average of 4 acres • Households with perceived security had more crop diversity compared with those that perceived themselves land tenure insecure • Consumption of vegetables and fruits (either vitamin A rich or not) was significantly higher among households that considered themselves land tenure insecure compared with the land tenure secure • Daily disposable income = positive correlation with dietary diversity • Land tenure insecurity = negative correlation with dietary diversity	X				X
Nikuze et al. [74] (2019). Livelihood impacts of displacement and resettlement on informal households – a case study from Kigali, Rwanda.	• Urban development and disaster-induced slum displacement → lack of income from previous rental properties • Loss of employment increased after resettlement		X		X		• Reduced food consumption and changed diets by buying cheap food products → reduced nutrition • Increased cost of transportation to urban markets, high cost of locally available food and loss of income increased the risk of eating only once a day		X			X
Potts et al. [49] (2019). Animal source food consumption in young children from four regions of Ethiopia: association with religion, livelihood, and participation in the productive safety net program.	• Pastoralists vs. agro-pastoralists vs. agriculturalist/farming households	X					• Children from pastoralist households were the most likely to have consumed animal-source foods in the preceding 24 h as compared with those in agropastoralist households or those in agriculturalist/farming households					X
Bonye et al. [45] (2021). Urban expansion and agricultural land use change in Ghana: implications for peri-urban farmer household food security in Wa Municipality.	• Conversion of agricultural land to activities of urbanization		X				• Declining farm productivity, partially caused by limited land availability for farming activities because of urban sprawl. • Major staple food crops such as yam, maize, cowpea, groundnut, and cassava, which initially was produced in abundance to feed the municipal population → inadequate to feed their families • Less food to feed their households → most of the food respondents ate over the last 12 mo were unbalanced	X				X
Manlosa et al. [72] (2019). Livelihood strategies, capital assets, and food security in rural Southwest Ethiopia.	• Smallholder farming households diversify livelihood strategies primarily through having a mix of food crops for subsistence, in combination with cash crops for income				X		• Increasing cash crop and commercially oriented food crop production • Households undertaking livelihood strategies with a higher number of food crops were more food secure than those with a lower number of food crops	X				
Matitia et al. [73] (2022). Subsidizing improved legume seeds for increased household dietary diversity: evidence from Malawi’s Farm Input Subsidy Programme with implications for addressing malnutrition in all its forms	• Enrollment in the Malawi Farm Input Subsidy Programme				X		• The cultivation of maize allows households to achieve energy food requirements, and although this fails to enhance diet diversity, our results suggest that those households that did achieve a surplus of maize were then able to make the choice to diversify their production into legumes	X				X
Asia (*n* = 24)												
Gironde et al. [55] (2021). No cash, no food. Gendered reorganization of livelihoods and food security in Cambodia.	• Subsistence agriculture → commercial agriculture • Agriculture → wage labor	X	X	X	X	X	• Reduced importance of rice as the main self-produced staple, decreased food collected, hunted and fished from common natural resources, and an increased share of food purchased in replacement of self-produced food • Incomes from cash crops and wage labor do not match the growing need for cash, whereas the increased cumulated workloads of females force families to compromise on food purchases and food preparation • As the number of days in wage employment decreases, the number of households reporting insufficient food rises • When cash income is lacking, females have to accommodate the meals. They cut either the number of meals per day, or the composition of the meals.	X	X	X	X	X
Gurung et al. [56] (2016). Transformation from rice farming to commercial aquaculture in Bangladesh: implications for gender, food security, and livelihood.	• Rice farming → commercial aquaculture farming (CAF) • Seasonal off-farm activities → low-paid nonfarm activities (pulling rickshaws, driving vans, and working as nonfarm wage labor)	X	X	X	X	X	• Rice production → commercial aquaculture farming • Females said access to and control over rice and fish for consumption at home had declined. They depended more on the market and on their husbands for the family food needs because the household income is controlled solely by males • Reduced intrahousehold bargaining power for food acquisition—wait for the harvesting period or husbands to procure fish or shrimp for the family meals	X	X			X
Euler et al. [78] (2017). Oil palm adoption, household welfare, and nutrition among smallholder farmers in Indonesia.	• Rubber farming → oil palm farming	X	X	X	X		• Local food markets are relatively well developed, so that a wide diversity of food items are available all year round, which has improved dietary quality as oil palm farmers make higher incomes and can provide better foods for their households		X			X
Alston and Akter [53] (2016). Gender and food security in Bangladesh: the impact of climate change.	• Agriculture → informal wage work (construction for males, garment factories for females) • Rural–urban migration • Shift from farming certain crops to other crops more resilient to climate change	X	X	X		X	• Impact of climate-related changes across regions—ability to grow food is diminished; less fresh food; quality of the food that is available is reduced; less variety of food; and more pressure to earn income to buy food • Seasonal food insecurity led to a greater reliance on the marketplace to purchase food, making income-generating activities or the taking out of loans a critical aspect of food security • When food is scarce, females and girls will eat less and that this will facilitate intrahousehold consumption smoothing	X	X		X	X
Sahu [83] (2018). Household drought coping, food insecurity and women in Odisha.	• Drought → shortfall in income forcing male migration for work • Females assume more significant share of the work burden by working extended periods and undertaking more tasks		X	X	X	X	• Shift from preferred foods to cheaper substitutes • Cutting quantity of meals, reducing the number of meals per day, favoring certain household members over others, and skipping meals • Reduced dietary intake is a common coping approach of drought-affected females as they try to moderate the impact on their families		X		X	X
McCarthy [57] (2019). The paradox of progressing sideways: food poverty and livelihood change in the rice lands of outer island Indonesia.	• Households “move sideways” by holding together “a complex and diverse array of temporally contingent and opportunistic network of activities” • “Truncated agrarian transition” = sites of production in agriculture and labor fail to provide sufficient opportunities • Many seek to escape poor agricultural livelihoods through wage labor and migration	X	X		X		• Most household production and consumption is exchanged through markets • Land ownership diminished, ensuring that farming households have less surplus production, and less rice to eat/sell • Cutting back on protein consumption during periods of scarcity • The poor spend approximately the same amount on snacks and tobacco as they do on rice and fish	X	X			X
Choithani [77] (2019). Gendered livelihoods: migrating men, left-behind women and household food security in India.	• Male's movement from agriculture → nonfarm/informal/wage work • Rural–urban migration • Females’ shift from homemaker to agriculture	X	X		X		• In households where females had some control over money, spending on food was prioritized • When their husbands were outside the village, they went to local markets to buy fresh/seasonal vegetables but they did not venture out of the house when their husbands were home		X			
Rowland et al. [29] (2022). Oil palm and gendered time use: a mixed-methods case study from West Kalimantan, Indonesia.	• Swidden farmers → off-farm labor on oil palm plantations	X		X	X	X	• Both males and females allocate more time to wage work and less time to agricultural production • Income from oil palm is insufficient to allow households to purchase an entire family’s food supply, so livelihood diversification is necessary • Purchasing and consuming preprepared foods from mobile vendors • Reduced consumption of foods that take longer to cook (meat) • Females’ evenings are primarily filled with domestic activities such as preparing the next day’s breakfast • Females working → payment for daycare and females leave food with workers to feed children	X	X	X	X	X
Sousa et al. [60] (2022). Patterns of street food purchase in cities from Central Asia.	• Rural–urban migration for work (e.g., street food customers are usually urban workers but rural or periurban dwellers, who often need to make long commutes between house and the workplace on a daily basis)		X	X	X		• Purchases consisted of a diverse set of both local and westernized foods and beverages, and varied throughout the day and by city location • Street food is often consumed as a substitute for home-cooked main meals, and may be closely associated with changes in time allocation • Industrial foods and beverages may be commonly preferred by the street food customers as mid-morning or mid-afternoon snacks (supply energy needs)		X			X
Tamrakar et al. [86] (2020). Drivers of healthy eating in a workplace in Nepal: a qualitative study.	• Agricultural → urbanized work (specifically hospital worksite)	X	X				• Unavailability of healthier food options in workplace canteen • Lack of necessary human resources to prepare more healthy food options in the workplace			X		X
Wertheim-Heck and Spaargaren [87] (2016). Shifting configurations of shopping practices and food safety dynamics in Hanoi, Vietnam: a historical analysis.	• Farmers have been pushed out of the city by land appropriation for urban development • Agriculture is looked down upon compared with occupations outside of agriculture	X	X				• A trend of buying online from farmers without direct personal contact has been observed • Buy from “neighboring” periurban farmers	X	X			
Steenbergen et al. [61] (2017). Effects of rapid livelihood transitions: examining local co-developed change following a seaweed farming boom.	• Dependence on diverse low-productivity livelihood activities → predominant focus on seaweed farming	X			X		• Increase in seaweed production from 2006 to 2010 then it has steadily decreased • Decrease in fishing and copra production with increase in seaweed cultivation • Consumption of instant noodles was often said to have been substituted for proper meals being cooked for younger family members • Higher income from seaweed farming led to more cash-based consumption patterns	X				X
Seng [59] (2015). The Effects of nonfarm activities on farm households’ food consumption in rural Cambodia.	• Shift to nonfarm activities	X			X		• Positive gains in per capita food consumption • Because nonfarm employment generates supplementary household incomes, it can provide participants with additional capital for investments in agricultural technology, which is a productivity-enhancing factor	X				X
Ashley et al. [54] (2018). Livestock and livelihoods of smallholder cattle-owning households in Cambodia: the contribution of on-farm and off-farm activities to income and food security.	• Diversifying income through smallholder cattle raising and off-farm activities	X			X		• In addition to cattle raising, households also reported producing fruit (75%), wet season rice (70%) and nonrice crops (60%) • Rice cultivation and poultry raising were considered valuable for household food requirements with product retained for human (milled rice and chicken meat) and animal consumption (rice meal and rice straw)	X				X
Piple et al. [82] (2015). Food choices and consequences for the nutritional status: insights into nutrition transition in an hospital community.	• Housekeeping vs. staff nurse vs. doctors	X			X		• The large number of underweight children among housekeeping families suggests that economic constraints limit calorie intake • There were more overweight children among nurse households compared with doctors and they consume more calories, protein and fat. Many of the nurses moved up from housekeeping and the author speculates that these mothers originally from poor backgrounds may tend to overcompensate, so that their children do not suffer the deprivations they suffered in their childhood.					X
Tran and Vu [62] (2020). The pro-poor impact of non-crop livelihood activities in rural Vietnam: a panel data quantile regression analysis.	• Households switching from a crop livelihood to any noncrop livelihood (e.g., livestock, wage-earning, nonfarm, private or transfer livelihoods)	X			X		• Increased food consumption					X
Morrison et al. [80] (2018). Formative qualitative research to develop community-based interventions addressing low birth weight in the plains of Nepal.	• Traditional household vs. nuclear household (male migrant workers)		X			X	• Those that worked in traditional households usually had first choice on what was cooked and eaten first • Work inside home was seen as less strenuous so they should eat less • More flexibility in food allocation, decision making and traditions in nuclear households				X	X
Gibson et al. [79] (2021). Coping or adapting? Experiences of food and nutrition insecurity in specialised fishing households in Komodo District, eastern Indonesia.	• 43% of households pursued 2 or more income-generating livelihood activities (typically home-based activities such as making and selling traditional cakes) • Females working				X	X	• Times of food insufficiency because of livelihood alterations → females prioritize other members of their household before feeding themselves, demonstrating inequality in intrahousehold distribution of nutrient-dense meals • Changes in diet and increasing short-term food supply → purchased cheaper and less-preferred foods • Typical household dietary pattern comprised frequent consumption of the staple rice and regular consumption of fish, and only occasional consumption of vitamin A rich foods and fruits and vegetables • Food insecurity higher in the wet season because of poor weather conditions which kept fishers ashore → less fish harvested for subsistence consumption or sold for income		X		X	X
Naz et al. [58] (2020). Women's contribution in provision of household food security: a study from rural areas of Punjab, Pakistan.	• Females participate in farming, labor force, and income-generating activities				X	X	• Females performed a substantial role in food production • Households with diverse sources of income were supplementary food secure as compared with single-source income households • Majority of rural households depend on their own food production → reduces dietary diversity • Households with livestock and poultry ownership had better food consumption scores and dietary diversity • 75% of females were preserving food such as drying vegetables, meat, making of jam, pickles, etc. for their households to keep food available for all times • When rural females having decision-making power at the household level and household economic resources were controlled and managed by females then households had a greater capacity to sustain a good food intake	X		X	X	X
Shukri et al. [84] (2018). Relationship between work-family conflict and unhealthy eating: does eating style matter?	• High workload, increased shiftwork and low payment rates • Human services, industry, and small businesses			X		X	• Unhealthy eating was positively correlated with work-to-family conflict, family-to-work conflict, emotional eating and external eating					X
Wertheim-Heck and Raneri [63] (2020). Food policy and the unruliness of consumption: an intergenerational social practice approach to uncover transforming food consumption in modernizing Hanoi, Vietnam	• Time-constrained modern lifestyles of females working out-of-home			X		X	• Discrepancy between the older and the younger females regarding their perceptions on how supermarkets fit within their daily life. The elderly females were more positive about the future potential of the convenience appeal of supermarkets than their daughters (in-law)		X			
Surendan et al. [85] (2020). Characterising the fruit and vegetable environment of peri-urban Hyderabad, India.	• Occupational shifts in India away from agriculture, with people now working in poultry farms and as manual laborers	X					• Loss of food from own production and increased acquisition and consumption of “outside foods”	X	X			X
Mottaleb et al. [81] (2017). Consumption of food away from home in Bangladesh: do rich households spend more?	• Females in nonfarm employment		X				• Females consume and spend more on food away from home, and spend more on food items than others		X			X
Central and South America (*n* = 3)												
Cleary et al. [64] (2022). Changes in food consumption in an indigenous community in southern Belize, 1979-2019.	• Rural–urban migration • Agriculture → nonfarm/informal/wage work • Young males spend increasing parts of the year “jobbing out”—working for wages in the cities in sectors such as construction, tourism and shrimp farms, then returning to the village to harvest corn and live with their families → become semiproletarians (wages supplement the subsistence they derive from the land)	X	X	X	X	X	• Purchased “outside” foods because of growth of cash economy and lack of time to obtain and prepare the preferred “local” foods • As semiproletarianization becomes increasingly frequent, new tastes for urban foods such as fried chicken, rice, and beans are acquired by males working outside the village, who, upon their return home, incorporate them into village life • Children who now attend high school outside the village are also exposed to new foodstuffs and food habits among their nonindigenous peers. Chicken, rice, and beans are aspirational dishes, signs of urban sophistication and national identity in comparison with foods from the “bush”		X	X		X
Oddo et al. [66] (2018). Pathways of the association between maternal employment and weight status among women and children: qualitative findings from Guatemala.	• Homemaker → wage work or other nonfarm income-generation activities	X	X	X	X		• Changes in food purchase after being employed. Mothers used additional income to purchase more meat and dairy, energy-dense food for households, often prioritizing their children’s requests. Exposure to take-away or street foods was more common for employed mothers compared with nonemployed ones • Changes in the time allocated for household duties as a result of employment included a decrease in time spent on meal preparation and food procurement—the grandmother often helped with meal preparation, childcare, and food procurement in mother’s absence • Nonemployed mothers also perceived that they would have less time to prepare meals or go to the market if employed		X		X	X
Kurschner et al. [65] (2020). Impact of school and work status on diet and physical activity in rural Guatemalan adolescent girls: a qualitative study.	• School → employment	X		X			• Eating schedule disruption • Meal skipping • Purchased processed foods but only for special occasions to share at home with rest of family					X
Mixed regions (*n* = 2)												
Banerjee et al. [67] (2015). A multifaceted program causes lasting progress for the very poor: evidence from six countries.	• Shift to self-employment strategies	X			X		• Per capita consumption increased					X
Diehl et al. [68] (2019). Household food consumption patterns and food security among low-income migrant urban farmers in Delhi, Jakarta, and Quito.	• Rural–urban migration—specifically from rural agriculture (more subsistence) livelihoods to urban agriculture (more commercial) livelihoods		X		X		• India: Households saved money to buy food at the market during the monsoon rain season and bought less food during that time. If the land flooded, households moved into government provided tents with food staples provided but reported as low quality • Indonesia: Occasional harvest failures during the monsoon season, but main source of household food was from markets and there was no change in dietary intake because of seasonality. The income generated from agricultural practice was enough to support food purchases • Ecuador: Consumed food from home gardens → not have to purchase as much from local markets, which saved them money each month. Individuals used money they saved to purchase better quality foods and food they could not afford previously, such as meat or fruit	X	X			X

Abbreviations: A, acquisition; C, consumption; D, distribution; I, income; L, locality; O, occupation; P, production; Pr, preparation; SR, social relation; T, time.

### Occupation

Occupations shift when individuals transition job or profession, or engage in multiple jobs (diversification), affecting production, acquisition, preparation, distribution, and consumption of foods. Of the 53 articles that were reviewed, 37 reported a change in occupation, resulting in a change in production (*n* = 17), acquisition (*n* = 21), preparation (*n* = 12), distribution (*n* = 9), and consumption (*n* = 32). Findings for production included shifts to nonfarm livelihoods being closely associated with reduced food production [27,42,50,57,88]. Increased market engagement and shift to market-oriented crops changed diets. Changes in commercial agricultural engagement—such as increased livestock production in Cambodia and Zambia [48,54], aquaculture farming in Bangladesh [56], or cash crops in Vietnam, Cambodia, Tanzania, and Indonesia [28,55,61,63]—led to shifts in the quantity and types of foods produced for commercial compared with household use. Without own food production, people depended on traditional and modern retailers to acquire food [27,53]. With increased dependence on food markets, households changed their preservation or storage techniques, allocation practices, and dietary patterns [28,29,53,58]. Overall consumption of kilocalories and diversity of foods increased when individuals shifted from traditional agricultural livelihoods to nonfarm livelihoods or self-employment [59,62,67]. Shifting from subsistence agriculture to aquaculture or small livestock production increased the consumption of animal-source foods [48,49]. In contrast, shifting from farming to nonfarming occupations decreased the consumption of traditional staples and animal-source foods and increased the purchase and consumption of ultraprocessed, convenience foods [28,46].

For example, in rural Indonesia, shifting from traditional agriculture or hunter–gatherer livelihoods to oil palm production shifted food choice behaviors [29]. Both males and females reported allocating more time to oil palm production and less time to agricultural production compared to recent years, resulting in purchasing and consuming preprepared foods from mobile vendors. As females spent more time working outside the home, their evenings often involved performing domestic activities, such as preparing the next day’s breakfast. With their new occupation, females also reported leaving their children at daycares, where workers fed their children, rather than being solely responsible for the child’s food allocation. Overall, shifting to oil palm production negatively impacted food consumption patterns with an increase in unhealthy and economically unsustainable foods [29].

### Locality

Locality is determined by the physical location of the workplace, which sometimes changes, such as through migration from a rural work environment (e.g., mines and farms) to an urban work environment (e.g., factories and street food stalls). Characteristics of localities have also changed in recent years with urbanization converting rural agricultural spaces into urban built environments [10]. Of the 53 articles that were reviewed, 26 reported a change in locality, resulting in a change in production (*n* = 9), acquisition (*n* = 21), preparation (*n* = 7), distribution (*n* = 9), and consumption (*n* = 23). Migration can be voluntary, in which individuals seek improved economic opportunities, or forced, due to danger, legal injunction, or external circumstances. Voluntary and forced migration typically reduced individual and household agricultural production [27,55,57,68]. Voluntary migration led to increased food availability and accessibility in a new food environment, affecting food acquisition and preparation behaviors and, subsequently, diets [46]. Lengthy work commutes and physically demanding occupations increased demand for mobile street food vendors near workplaces [30,44,51,60,64,66].

For example, young males in Belize who migrated to cities for informal wage work developed aspirations for modernity, having experienced different urban lifestyles and cultures; these new aspirations sometimes shifted diets away from traditional foods and toward Western or convenience foods prepared outside the home, such as fried chicken [64]. Once these workers returned to their villages, they introduced urban tastes into their rural families’ diets.

Migration because of urbanization impacting land use was also associated with negative changes in food choice behaviors. For example, in Ghana, urban sprawl limited arable land available for agricultural activities, resulting in lower farm productivity. This forced households to reduce food consumption and dietary diversity, ultimately increasing household food insecurity [45].

### Time

Time available for food-related behaviors shifts with livelihood changes. For example, some livelihood changes require longer and less flexible working hours, or longer commutes. A change in time use was reported in 23 of the articles reviewed, resulting in changes in production (*n* = 7), acquisition (*n* = 16), preparation (*n* = 10), distribution (*n* = 11), and consumption (*n* = 20). As individuals shifted to nonfarm and off-farm activities, less time and effort was spent on household agricultural production, decreasing individual production or increasing hired wage labor to conduct on-farm activities [27,29,30,42,55,56]. With reduced time available for production, households acquired more food outside the home [27,44,46,60,64,66]. Despite the perception that foods prepared from scratch were healthier, the real or perceived time required to prepare these foods resulted in the procurement of ready-to-eat foods (e.g., street foods, fast foods, and snacks) or those that were quicker and easier to prepare (e.g., instant noodles and processed meats) [46,64,66]. More time spent commuting to and from workplaces and longer work hours disrupted eating and food distribution schedules and was associated with skipped meals or negative changes in the composition and quality of meals (e.g., consuming preprepared foods) [29,44,55,65].

For example, in Mozambique, females’ participation in wage work in addition to farming created time constraints for food preparation and consumption, which reduced the number of meals consumed or led to repetition of the same meals at different mealtimes within a day [52]. The study reported that in the past month, 43% of respondents skipped a meal because of the lack of time to prepare it and 65% reported that someone in their household had rejected a meal to avoid eating the same food for both lunch and dinner [52].

### Income

Income changes refer to a shift in financial compensation when individuals or households adopt a new livelihood (e.g., wage work to self-employment). A change in income was reported in 39 articles, resulting in alterations to production (*n* = 19), acquisition (*n* = 24), preparation (*n* = 11), distribution (*n* = 11), and consumption (*n* = 34). As cash-generating activities increased, food production decreased, and people depended more on food markets [27,28,42,50,55,57,68].

Affordability is a key food choice consideration, and unstable employment, particularly in the informal sector, may impact how much and what types of foods were purchased, prepared, distributed, and consumed [30]. Higher incomes from livelihood change were associated with investments in not only more nutrient-dense foods, such as animal-source foods, fruits, and vegetables [12,27,47,66,68], but also energy-dense, nutrient-poor foods, such as sugar-sweetened beverages or biscuits [46,51,52,66].

In West Africa’s artisanal and small-scale mining sector, there was high earning potential, but the lack of guaranteed daily compensation affected food choice [30,47]. In northeastern Guinea, females shifting to artisanal and small-scale mining obtained limited food from self-production and were largely dependent on markets. They often selected vendors with whom they could cultivate a relationship, enabling food purchasing on credit. These arrangements were critical for miners who faced income instability [27]. Miners typically used their earnings to purchase fish, meat or poultry, vegetables, and cooking oil, but these nutritious food purchases were contingent on the productivity of the day’s labor [27]. Similarly, female miners routinely purchased food upon returning from the mining sites; therefore, what was prepared for the evening meal reflected the miner’s daily success [30]. If income was lacking, females might skip meals themselves to ensure enough food for their families.

### Social relations

Social relations create dynamics around food behaviors [89]. Changing gender roles and interpersonal connections, including more females working outside of the home, exposure to global media, and interactions with different cultures, may affect food choice behaviors. Changes in social relations were reported in 21 articles, resulting in a change in production (*n* = 8), acquisition (*n* = 15), preparation (*n* = 11), distribution (*n* = 12), and consumption (*n* = 18). Females’ involvement in on-farm occupations increased food production [58], whereas females’ involvement in off-farm occupations decreased production [28,29,55]. Females’ involvement in paid labor was associated with increased decision-making power, positively affecting food acquisition and distribution, and usually improved the household’s dietary diversity. However, results were mixed for food preparation practices: some studies reported no changes in food preparation practices, whereas others reported a negative shift to quicker, convenience foods [43,58]. In urban areas, exposure to global eating patterns, modern mass media, and formal and informal nutrition knowledge led to changes in food preferences and habits, ultimately increasing acquisition and consumption of ultraprocessed foods and meals away from home [28,44,46,64].

For example, in rural Guatemala, mothers who shifted from homemaking to working outside the home changed their food purchases to include more meat and dairy, increasing food quantity and variety [66]. Several mothers also purchased “indulgent,” energy-dense foods and reported greater exposure to take-away and street foods compared with nonemployed mothers. Some mothers also enlisted their mothers or mothers-in-law for food acquisition, preparation, and distribution practices. Other females discussed challenges delegating domestic tasks to other household members, leading them to rely on convenience foods to feed their families because of time limitations and accumulation of other household tasks [66].

## Discussion

This review identified and characterized the major mechanisms through which livelihood changes affect food choice behaviors: occupation, locality, time, income, and social relationships. Food production behaviors were affected by livelihood changes, particularly shifting to market-oriented occupations; this process altered the balance of the availability and affordability of foods in local food environments compared with own food production. Acquisition behaviors were heavily influenced by all mechanisms but especially by changes in income that impacted the types of foods purchased. Locality changes affected the types of foods available for purchase as well as the quantity and quality of wild and/or indigenous foods available. Changes in time use influenced when and how individuals were able to acquire foods. Preparation behaviors were sensitive to time limitations induced through livelihood change that affected the amount of time available for preparing meals, whereas changes in location altered food preparation methods. Food distribution and consumption behaviors were also heavily influenced by livelihood-induced time limitations, with the need for convenience coupled with higher discretionary incomes being key drivers of processed and packaged food consumption. Altered social relationships and social norms were a consequence of livelihood changes that led to expansion of food preferences to include nontraditional food consumption, particularly among urban dwellers.

Global changes in economic process, migration, urbanization, food environment changes, climate change, and crises such as the SARS-CoV-2 pandemic and ongoing wars have corresponded with large-scale changes in food supply chains and food choices resulting in dietary patterns moving toward ultraprocessed foods that contain high amounts of refined carbohydrates, sodium, and saturated or transfats [[90], [91], [92]]. Livelihood shifts in types of occupation, work conditions, and work environments have shown some associations with increased reliance on ultraprocessed foods [93]. Females working outside the home and alterations in gender roles substantially changed time use available for food choice and dietary patterns. The consumption of ultraprocessed foods contribute to poor health, including overweight, obesity and diet-related noncommunicable diseases, and environmental harms, with increased greenhouse gas emissions, loss of biodiversity, and environmental degradation from food production [94].

Time limitations appeared to contribute to reliance on unhealthy foods such as preprepared, ultraprocessed, convenience foods, leading to various negative health consequences, including the widespread adoption of unhealthy food choices [[95], [96], [97], [98], [99]]. Unhealthy eating patterns can result from an emphasis on convenience related to caregiver time limitations, ease in access to ultraprocessed foods, and exacerbated by further livelihood changes, with implications that are intergenerational when children’s early life food exposures and diets are suboptimal. A literature review on factors contributing to childhood malnutrition found that an increase in maternal employment may increase occupational time demands, potentially limiting their ability to provide sufficient care for their children [100]. This, in turn, may be associated with child malnutrition [100].

Similar to high-income countries, people in LMIC are demanding foods with shorter and simpler steps to prepare, minimal ingredients, and which can be made in advance and preserved or stored for long periods [41,46,66,101]. When faced with a choice between maintaining traditional food practices or purchasing affordable foods that free up time, findings from the review suggest that many households will choose the latter because of livelihood changes. Successful campaigns to promote healthy traditional food consumption must incorporate the value of time for their target populations. For example, in India, a campaign to promote millet consumption to improve food security and health acknowledges that preparation of the grain is very time intensive and may not appeal to households with constraints on time or facilities [102].

Locality shifts related to livelihood changes are heterogeneous. For instance, 1 family member may migrate to an urban area to engage in wage work, whereas the remaining members remain in the villages and continue to maintain the family farm or other rural business [12,103]. In other instances, individuals or households keep their rural residence and commute daily to an urban or periurban area for work [104]. Others move their entire family unit from a rural to urban or periurban environment [23]. These locality shifts may have different consequences for food choice behaviors and healthfulness of diets, particularly depending on proximity to markets and greater commercialization [105].

Out-of-home livelihood opportunities for females can increase empowerment through higher incomes, access to information and resources, and expansion of social networks. Females who shift from homemaking to employment outside the home often have more decision-making power for household food acquisition and consumption [[106], [107], [108], [109]]. Less time for home food preparation, however, can decrease dietary diversity and increase reliance on ultraprocessed foods, especially if another household member does not take on some of the burden of food acquisition, preparation, or distribution. Interventions that target household, maternal, or child nutrition may be more successful if they address household food roles and shared responsibility directly. An example of an intensive females’ empowerment intervention focused on improving employability of females achieved positive impacts on food security and weight status through education, self-esteem and self-efficacy promotion, and changes in gendered behaviors related to coresponsibility and self-care [101].

Recognizing the vital intersection of livelihood change and dietary health, interventions and policies aimed at effectively addressing these dynamics are of great importance. Some policies, however, can exacerbate the negative impacts of livelihood changes on healthfulness of diets. For example, policies attempting to address food safety concerns by eliminating the availability of informal food outlets (e.g., wet markets and open-air markets) and building modern food outlets (e.g., supermarkets) can have unintended consequences by reducing healthy food access and compromising the livelihoods of informal sector workers those who are both vendors and consumers [63]. Informal food retailers were particularly adversely affected by the COVID-19 pandemic, with closure of markets resulting in loss of income and amplifying risks of food insecurity [110]. Because of the lack of proper guidance and implementation in other informal sector occupations, the issue of worker exploitation continues to present challenges, as these occupations are “under the table” and workers typically do not receive benefits, injury protection, or job security, which can hinder the ability to access nutritious foods and healthcare [7].

This is the first review to the best of our knowledge to synthesize research on livelihood change affecting food choice behavior changes. The goal is for this work is to guide future research in this area, which is increasingly important with a likely increased frequency of livelihood changes in LMIC occurring with escalating climate change and other pressures. Only 3 articles in this review were from Latin America, highlighting the need for further research in this context. This could potentially be attributed to the reclassification of numerous countries in the region, which were historically categorized as lower-middle-income but have since been redefined as upper-middle-income economies. Because of the nature of a scoping review, this review did not include an assessment of the quality of the articles reviewed. There also may be additional relevant articles that were missed because of database selection, period (e.g., restricting article publications to those published between 2015 and 2022), or exclusion of articles published in a language other than English. A scoping review was deemed more suitable than a systematic review for this study because of its emphasis on mapping the breadth of available literature, identifying knowledge gaps, and facilitating a comprehensive understanding of livelihood change and food choice behaviors.

The designs and methods of the reviewed studies provide plausible evidence that the identified mechanisms operate in a causal manner. Although many of the studies were cross-sectional, many used mixed methods, important for data triangulation, and longitudinal methodologic types for which it is easier to infer causality. Most studies used a qualitative method that provided in-depth understanding of the relationship between livelihood change and food choice behaviors including the causal direction. Only 2 quantitative studies used randomized control designs, so future quantitative research that uses experimental or quasiexperimental designs would likely be fruitful, as would longitudinal research examining these mechanisms over longer time periods.

Five mechanisms—occupation, locality, time, income, and social relations—for how livelihood change affects food choice behaviors were identified (Figure 2). These livelihood changes lead to changes in individual food choice behaviors, including decreased household food production, increased acquisition of foods from markets, decreased preparation of food at home, altered household meal patterns, and increased consumption of ultraprocessed preprepared convenience foods. Understanding the changing patterns of daily life in LMIC amid livelihood changes can help ensure research recommendations for improving food choices are contextually grounded for target populations. A more detailed understanding of the mechanisms through which livelihood changes affect nutrition behaviors and outcomes could help to inform appropriate policy and programmatic levers for intervention to support vulnerable populations in achieving optimal health and well-being.

## Author contributions

The authors’ contributions were as follows – KKR, EAF, LIR, SB, SN, HW, SW-H, AI, SAC, RA, BE, MM, CEB: conceptualized the project; EK, KKR, SS, EAF, LIR, SB, SN, HW, SW-H, AI, SAC, RA, BE, MM, CEB: developed the research plan; EAF, CEB: provided study oversight; EK, KKR, SS, EAF, LIR, SB, CEB: conducted the research; MB, SN, HW, SW-H, AI, SAC, RA, BE, MM: provided essential materials; EK, KKR, SS, EAF, CEB: analyzed the results; EK, KKR, SS, EAF, CEB: wrote the paper; EK, EAF, CEB: had primary responsibility for final content; and all authors: reviewed and approved the final manuscript.

## Conflict of interest

The authors report no conflicts of interest.

## Funding

This research has been funded by the Drivers of Food Choice (DFC) Competitive Grants Program, which is funded by the UK Government’s Foreign, Commonwealth & Development Office and the Bill & Melinda Gates Foundation, and managed by the University of South Carolina, Arnold School of Public Health, United States; however, the views expressed do not necessarily reflect the UK Government’s official policies.
