# Supplementary material for: How Livelihood Change Affects Food Choice Behaviors in Low- and Middle-Income Countries: A Scoping Review
Source: Adv Nutr. 2024 Mar 9;15(5):100203. doi: 10.1016/j.advnut.2024.100203 (PMC11007434; doi:10.1016/j.advnut.2024.100203)
Supplement: Multimedia component 1 [file mmc1.pdf]

How livelihood change affects food choice behaviors in low- and middle-income countries: A scoping review
Emma Kenney

Supplementary Table 1: PubMed Search Strategy

Category	Line	Query
Location	1	"Africa"[MeSH Terms] OR "Asia"[MeSH Terms] OR "South America"[MeSH Terms] OR "Pacific Islands"[MeSH Terms] OR "Central America"[MeSH Terms]<br><br>OR<br><br>"lmic*"[Title/Abstract] OR "third world countr*"[Title/Abstract] OR "lami countr*"[Title/Abstract] OR "transitional countr*"[Title/Abstract] OR "developing countr*"[Title/Abstract] OR "low and middle income countr*"[Title/Abstract]
Livelihood change	2	"Unemployment"[Title/Abstract] OR "Workplace"[Title/Abstract] OR "Informal Sector"[Title/Abstract] OR "livelihood*"[Title/Abstract] OR "livelihood change*"[Title/Abstract] OR "livelihood diversification"[Title/Abstract] OR "employ*"[Title/Abstract] OR "work*"[Title/Abstract] OR "profession*"[Title/Abstract] OR "income source"[Title/Abstract] OR "income generating activit*"[Title/Abstract] OR "job"[Title/Abstract] OR "jobs"[Title/Abstract] OR "career*"[Title/Abstract] OR "vocation*"[Title/Abstract] OR "wage work*"[Title/Abstract] OR "nonfarm*"[Title/Abstract] OR "non farm*"[Title/Abstract] OR "off farm*"[Title/Abstract] OR "self employ*"[Title/Abstract] OR "on farm*"[Title/Abstract] OR "day labor*"[Title/Abstract]
Food Choice	3	"food choice*"[Title/Abstract] OR "food decision*"[Title/Abstract] OR "food motive*"[Title/Abstract] OR "food select*"[Title/Abstract] OR "food option*"
Consumption	4	"consum*"[Title/Abstract] OR "eat"[Title/Abstract] OR "eating"[Title/Abstract] OR "meal*"[Title/Abstract] OR "snack*"[Title/Abstract] OR "breakfast*"[Title/Abstract] OR "lunch*"[Title/Abstract] OR "dinner*"[Title/Abstract] OR "drink*"[Title/Abstract] OR "ingest*"[Title/Abstract]
Production	5	"produc*"[Title/Abstract] OR "farm*"[Title/Abstract] OR "cultivat*"[Title/Abstract] OR "harvest*"[Title/Abstract] OR "agriculture"[Title/Abstract] OR "crop rais*"[Title/Abstract] OR "grow*"[Title/Abstract] OR “breed*” OR “rais*”[Title/Abstract] OR “pastoral*” [Title/Abstract] OR “garden*”[Title/Abstract] OR “plant*”[Title/Abstract] OR “horticulture”[Title/Abstract]
Acquisition	6	"aqui*"[Title/Abstract] OR "buy"[Title/Abstract] OR "buying"[Title/Abstract] OR "market"[Title/Abstract] OR "shop*"[Title/Abstract] OR "purchas*"[Title/Abstract] OR "procur*"[Title/Abstract] OR "obtain*"[Title/Abstract] OR "get"[Title/Abstract] OR "ingredient*"[Title/Abstract]
Preparation	7	("prepar*"[Title/Abstract] OR "cook*"[Title/Abstract] OR "bake*"[Title/Abstract] OR "baking"[Title/Abstract] OR "boil*"[Title/Abstract] OR "fry"[Title/Abstract] OR "frying"[Title/Abstract] OR "grill*"[Title/Abstract] OR "heat*"[Title/Abstract] OR "chop*"[Title/Abstract])

Distribution	8	"distribut*"[Title/Abstract] OR "provision*"[Title/Abstract] OR "management"[Title/Abstract] OR "allocat*"[Title/Abstract] OR "supply"[Title/Abstract] OR "shar*"[Title/Abstract] OR "feed*"[Title/Abstract] OR "mealtime"[Title/Abstract] OR "meal tim*"[Title/Abstract] OR "ration*"[Title/Abstract] OR “intrahousehold food distribution”[Title/Abstract] OR “intrahousehold food allocation”[Title/Abstract] OR “infant and young child feeding”[Title/Abstract]
Combination(s)		Step 1: (1) AND (2) AND (3)<br>Step 2: (1) AND (2) AND (3) AND (4)<br>Step 3: (1) AND (2) AND (3) AND (5)<br>Step 4: (1) AND (2) AND (3) AND (6)<br>Step 5: (1) AND (2) AND (3) AND (7)<br>Step 6: (1) AND (2) AND (3) AND (8)
